# Intensity over duration in neurological rehabilitation: exploring evidence for optimised recovery paradigms

**DOI:** 10.3389/fneur.2025.1697186

**Published:** 2026-01-22

**Authors:** Ibrahim Npochinto Moumeni

**Affiliations:** 1 Department of Physical Therapy and Physical Medicine, Faculty of Medicine and Pharmaceutical Sciences, University of Dschang, Dschang, West Region, Cameroon; 2 Department of Physical Medicine and Osteopathy, Regional Hospital of Bafoussam, Bafoussam, West Region, Cameroon; 3 Institute for Applied Neurosciences and Functional Rehabilitation (INAREF), Odza-Yaoundé, Cameroon; 4 Franco-African Center for Applied Rehabilitation and Health Sciences (CFARASS), Foumbot, West Region, Cameroon; 5 Department of Geriatrics and Gerontology, Sorbonne Université, Pitié-Salpêtrière Hospital, Boulevard de l’Hôpital, Paris, France; 6 Private Practitioner, Paris, France; 7 Faculty of Health Sciences, University of Parakou, Parakou, Benin; 8 French-Speaking African Society for Neurorehabilitation (SAFNeR), Parakou, Benin; 9 UREKIM – Research Unit in Physiotherapy and Physical Medicine, Faculty of Medicine and Pharmaceutical Sciences, University of Dschang, Dschang, West Region, Cameroon; 10 Centre de Recherche en Santé Humaine et Développement des Médicaments (CRESHDEM), Faculty of Medicine and Pharmaceutical Sciences, University of Dschang, Dschang, West Region, Cameroon

**Keywords:** muscle plasticity, neuroplasticity, rehabilitation intensity, repetition density, stroke recovery, therapeutic dose, family-based rehabilitation, intensity-dependent plasticity

## Abstract

**Background:**

Contemporary stroke rehabilitation protocols traditionally emphasise session frequency and treatment duration over intervention intensity—yet emerging evidence suggests we may be preparing patients for therapeutic marathons when their brains demand neuroplastic sprints. Across neuroscientific, behavioural, and clinical domains, convergent data indicate that repetition density, metabolic load, engagement, and temporal compression—not cumulative minutes—constitute the biologically meaningful drivers of neuroplastic and myoplastic adaptation.

**Objective:**

This Perspective re-examines current rehabilitation paradigms through an intensity-centred lens, synthesising mechanistic evidence, clinical trials, and cross-cultural implementation models to determine whether high-intensity paradigms can more efficiently exploit neuroplastic windows and muscle adaptation dynamics.

**Methods:**

Evidence was integrated from intensity-focused RCTs, high-repetition upper limb training, HIIT-based protocols, constrained-duration boot-camp models, and comparative observations from West-Cameroon intensive programmes. Mechanistic principles of threshold-dependent plasticity, critical timing windows, and therapeutic momentum were analysed alongside real-world feasibility data from low-resource systems.

**Results:**

Across studies and contexts, high-intensity protocols—4–6 h/day for 3–4 weeks—consistently produced functional gains equivalent or superior to those achieved through conventional 1–2 h sessions over 12–16 weeks. Both neural and peripheral muscle plasticity responded more robustly to concentrated stimulation than to prolonged low-density regimens. Family-integrated programmes amplified therapeutic density and sustained momentum, demonstrating that intensity can be achieved without advanced technology.

**Conclusion:**

Rehabilitation effectiveness depends less on session duration than on the biological potency of stimulation delivered per unit time. Intensity-centred models align more closely with known mechanisms of neuroplasticity and muscle adaptation, offering a more efficient, scalable, and context-responsive pathway to post-stroke recovery. Future research should formalise intensity indices, determine minimal effective thresholds, and evaluate phenotype-specific dosing strategies to support the evolution toward precision rehabilitation.

## 1 Introduction

The optimal dosage of rehabilitation for driving post-stroke recovery remains a central unresolved issue in contemporary neurorehabilitation. Conventional care relies primarily on session duration and weekly frequency as its main dosing parameters, despite converging evidence suggesting that these temporal indicators insufficiently capture the true determinants of neuroplastic change (1–3).

A growing body of work indicates that intensity—defined as the density of neurofunctional stimulation delivered per unit time—may act as a more potent driver of recovery than cumulative therapy hours alone. Meta-analyses evaluating augmented therapy time (4, 5), constraint-induced movement therapy (CIMT) trials (6), high-intensity sensorimotor and aerobic programs (7), and intensive “boot-camp” models such as the Queen Square Upper Limb Programme (8) consistently highlight that concentrated high-intensity stimulation yields superior functional outcomes compared to equivalent volumes distributed over extended periods.

This evolving understanding is further reinforced by observations from diverse healthcare systems. In several African and European rehabilitation settings, models built around short, dense, temporally compressed intervention windows have demonstrated functional gains often surpassing those achieved under conventional duration-based regimens (9, 10).

Despite these convergent signals, several key knowledge gaps persist. First, the field lacks a coherent operational definition of “rehabilitation intensity.” Second, existing conceptual frameworks remain fragmented, with limited integration across mechanistic thresholds, temporal windows of heightened plasticity, or momentum-based therapeutic dynamics. Third, the literature lacks a structured synthesis of trials directly comparing intensity-focused versus duration-focused strategies, particularly in studies published between 2018 and 2025. Fourth, the transposability of intensive paradigms to low-resource environments has rarely been analyzed through a systematic theoretical lens. Finally, the hierarchy of evidence—ranging from animal models to large-scale clinical trials—remains insufficiently articulated.

The present Perspective addresses these limitations by: (1) providing a precise operational definition of rehabilitation intensity; (2) synthesising contemporary evidence (2018–2025), including a structured comparison of intensity-versus duration-dominant trials; (3) proposing an integrated theoretical framework composed of three unified models governing intensity-dependent recovery mechanisms; (4) clarifying the hierarchical distinction between mechanistic, clinical, and implementation evidence; (5) analyzing the cross-cultural adaptability of intensive rehabilitation and the amplification effect of family-integrated practice; (6) identifying priority research gaps and hypothesis-driven avenues; and (7) outlining clinical implications for the emerging field of precision rehabilitation.

Through this approach, the article responds directly to recent methodological recommendations calling for clearer conceptualization, improved analytical integration, and more rigorous benchmarking of rehabilitation intensity in stroke recovery research (8–10).

### 1.1 Conceptual foundations: developmental, behavioural, and lesion-induced plasticity

A unified understanding of how rehabilitation intensity modulates recovery requires grounding current neurorehabilitation theory in a cohesive model of brain and muscle plasticity. As articulated by Npochinto Moumeni (5) and further conceptualised in his complementary work on muscle plasticity (2), adaptive recovery after stroke emerges from the continuous interplay of developmental, behavioural, and lesion-induced plasticity. These three systems form the biological matrix upon which therapeutic stimulation—especially when delivered at high intensity—exerts its effects.

Developmental plasticity establishes the primary neural architecture enabling later adaptive mechanisms. Although largely maturational, it remains relevant throughout life as the structural networks it shapes provide the substrate for post-injury reorganisation. This foundational system explains why certain circuits reorganise more efficiently, and why early-life plasticity leaves a functional imprint detectable even in adult rehabilitation trajectories (2, 5, 9, 10).

Behavioural plasticity, central to post-stroke rehabilitation, represents the experience-dependent capacity of the nervous system to reorganise in response to internal constraints (motivation, effort, salience) and external constraints (task structure, repetition, therapeutic intensity). In Moumeni’s framework, behavioural plasticity has two opposite trajectories: (1) positive behavioural plasticity, driven by high repetition density, enriched tasks, proprioceptive loading, and sustained engagement; and (2) negative behavioural plasticity, arising from non-use, immobility, pain, or insufficient stimulation. High-intensity rehabilitation acts primarily by biasing the system toward positive behavioural plasticity, thereby preventing the maladaptive “non-use spiral” frequently observed in chronic post-stroke motor syndromes. This duality mirrors analogous mechanisms in muscle plasticity, where targeted overload leads to favourable myofibrillar remodelling while under-use rapidly induces muscle atrophy, architectural disorganisation, and metabolic inefficiency (2, 5).

Lesion-induced plasticity refers to the spontaneous and guided reorganisation triggered by the vascular insult. It is most active during the first weeks to months after stroke, a period of heightened synaptic malleability, axonal sprouting, and cortical remapping. Moumeni’s 2021 synthesis highlights that this post-lesional reorganisation follows a predictable hierarchy: early recruitment of redundant pathways, compensatory activation of secondary networks, limited regenerative processes, and delayed anatomical repair. The therapeutic challenge is to convert spontaneous reorganisation into functionally meaningful reorganisation, a transition strongly accelerated when intensity is increased during these biologically permissive windows (2, 5).

Crucially, these three plasticity systems operate as a dynamic triad, with behavioural plasticity forming the active interface between structural potential (developmental) and biological opportunity (lesion-induced). High-intensity rehabilitation acts precisely on this interface: it amplifies behavioural plasticity, steers lesion-induced changes toward adaptive circuits, and reactivates dormant developmental pathways. The combined inclusion of brain and muscle mechanisms reinforces the notion that neuroplasticity and myoplasticity evolve as a single, integrated adaptive system, requiring dosing strategies that exceed minimal activation thresholds (2, 5).

**Figure 1:**
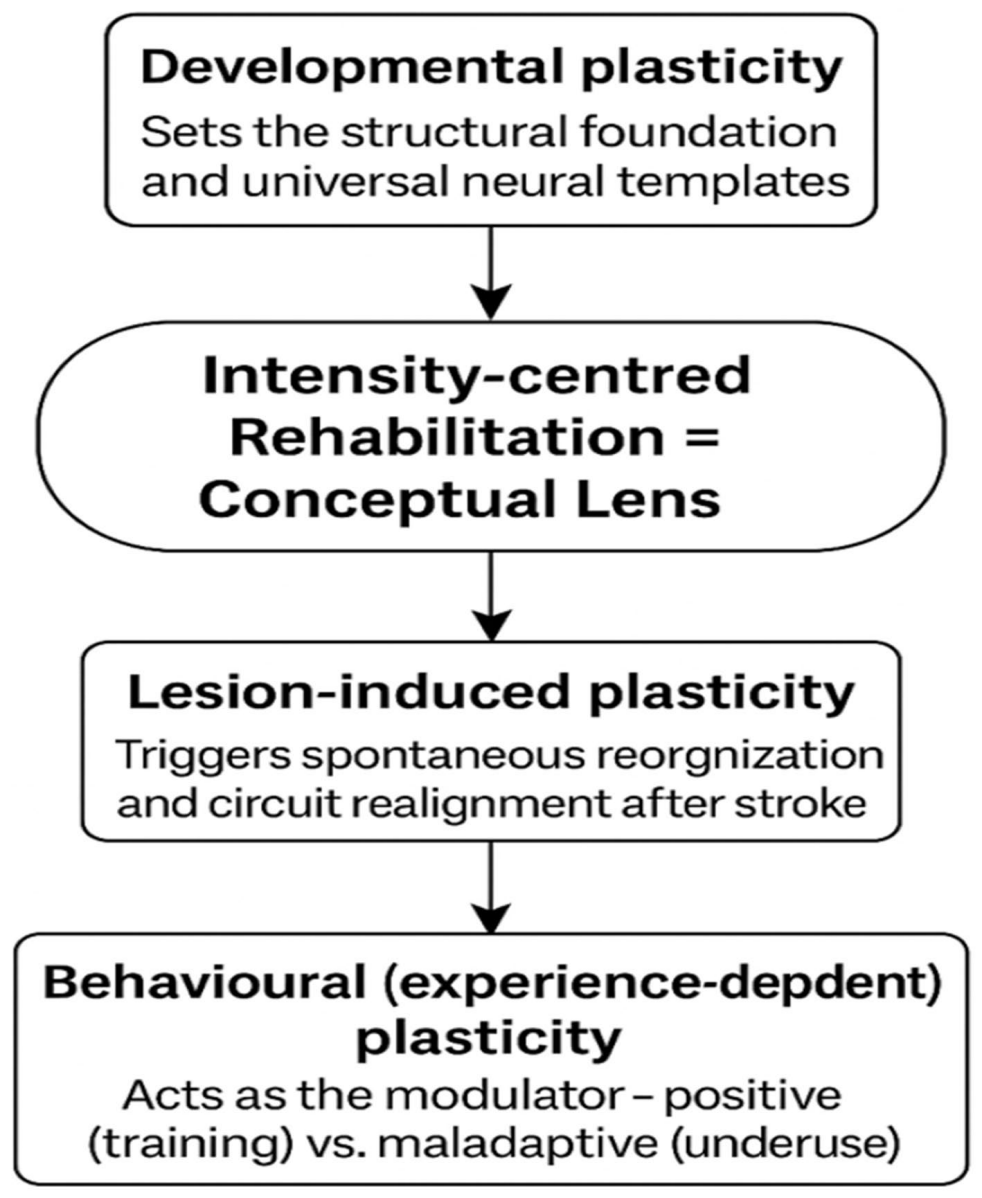
Brain plasticity framework supporting intensity-dependent rehabilitation. This conceptual model posits that intensity-centered rehabilitation acts as a lens to harness three interconnected levels of neuroplasticity: developmental plasticity setting the structural foundation and universal neural templates; lesion-induced plasticity triggering spontaneous reorganisation and circuit realignment after stroke; and behavioural (experience-dependent) plasticity acting as the modulator through positive (training) versus maladaptive (underuse) influences. Adapted from the neuroplasticity framework proposed by Moumeni (2).

Figure 1 synthesises these interactions and introduces the conceptual lens through which the remainder of this Perspective interprets the mechanisms and clinical implications of intensity-dependent rehabilitation.

## 2 Operational definition of “rehabilitation intensity”

The concept of rehabilitation intensity remains one of the most inconsistently defined constructs in neurorehabilitation science. Across the literature, “intensity” has been variously described in terms of session frequency, task repetitions, metabolic load, patient effort, attentional engagement, or the sheer volume of activities performed. This terminological fluidity has hindered the development of unified theoretical frameworks and has contributed to substantial methodological heterogeneity in clinical trials (11, 12).

To address these limitations, and in accordance with recent recommendations for standardisation in stroke recovery research (13), we propose a precise, operational, and multi-dimensional definition that aligns biological plausibility with clinical measurability.

### 2.1 Core definition

Rehabilitation intensity is defined as the rate at which neurofunctional stimulation is delivered per unit time, integrating four quantifiable components:

1. Repetition density: the number of purposeful motor, sensory, or cognitive actions performed per minute.
2. Neurophysiological load: the metabolic, cortical, and proprioceptive demands elicited during task execution.
3. Goal-directed effort and attentional engagement: the proportion of tasks performed near the patient’s maximal safe performance capacity.
4. Temporal compression: the degree to which stimulation is concentrated within therapeutic windows rather than distributed across extended periods. This definition distinguishes intensity from duration, volume, or frequency, thereby preventing inappropriate conceptual substitution.

### 2.2 Mathematical formulation

To facilitate reproducibility, we propose expressing intensity as:

$$\text{Intensity}=(R^{*}\;L^{*}\;E)/T.$$

where:

- R = repetition count per session
- L = neurophysiological load (scaled 0–10; metabolic + proprioceptive challenge)
- E = engagement/effort ratio (% of task execution performed above the adaptive threshold)
- T = session time in minutes

This formulation provides a basis for constructing standardised intensity indices, enabling comparison across trials and settings.

### 2.3 Clinical operationalization

Translating this definition into clinical practice requires practical measurement tools aligned with each component:

1. Repetition density can be quantified using task-specific counters, motion tracking devices, or standardised observational scales.
2. Neurophysiological load can be estimated using heart rate monitoring, perceived exertion scales, electromyography, or functional near-infrared spectroscopy.
3. Goal-directed effort and engagement can be assessed using patient-reported measures, therapist ratings, or validated engagement scales.
4. Temporal compression can be calculated as the ratio of active therapy time to total rehabilitation duration.

Integrating these measures into a unified intensity score provides a standardised method for quantifying and reporting intensity in both research and clinical settings. This operational framework aims to enhance methodological rigor, facilitate inter-study comparisons, and guide the design of intensity-optimised rehabilitation protocols.

**Figure 2:**
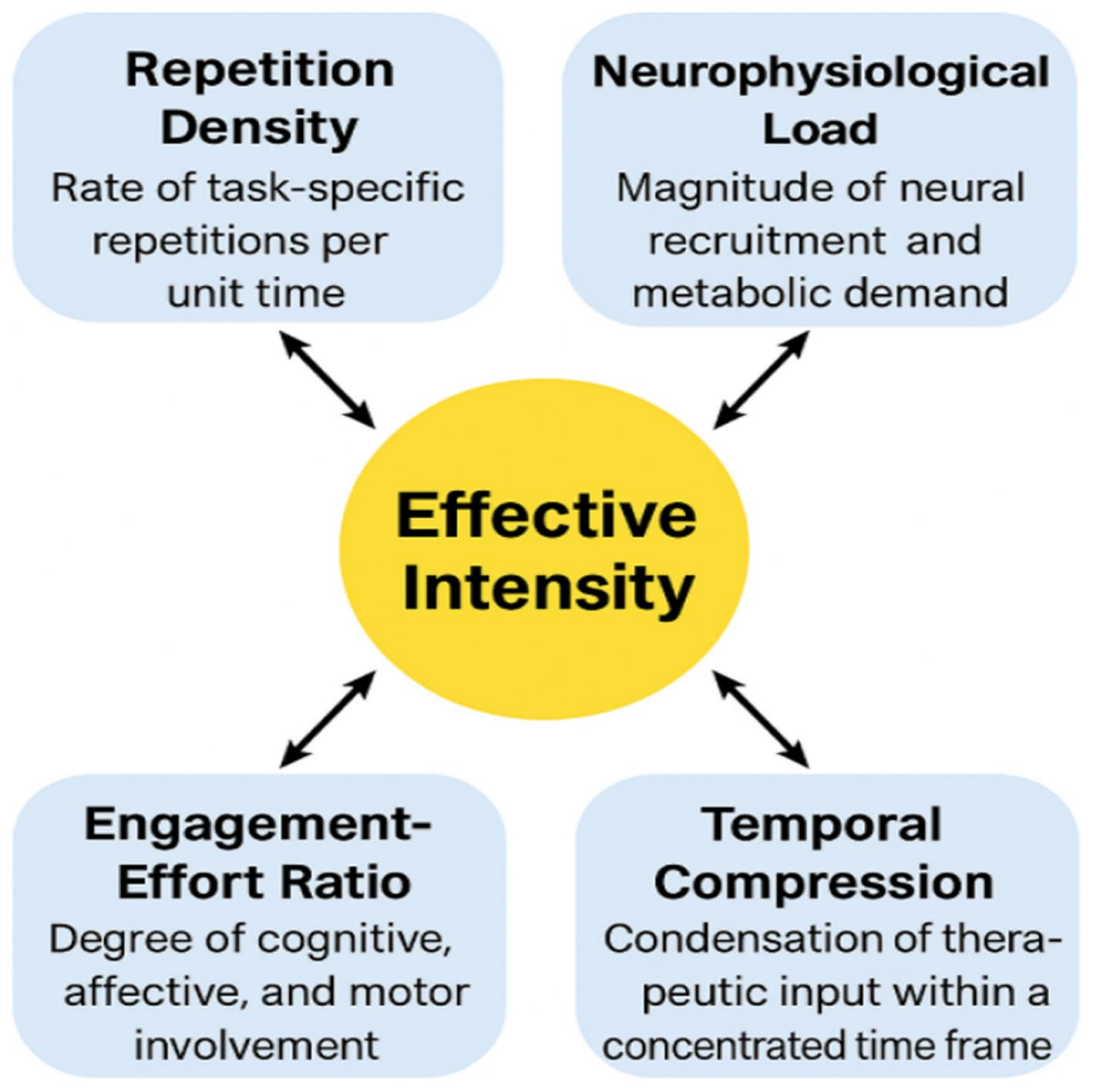
Multidimensional operational model of rehabilitation intensity. Adapted from Moumeni (9, 10). This conceptual model posits that effective intensity arises from the synergistic interaction of four quantifiable components: repetition density (rate of task-specific repetitions per unit time), neurophysiological load (magnitude of neural recruitment and metabolic demand), engagement–effort ratio (degree of cognitive, affective, and motor involvement), and temporal compression (condensation of therapeutic input within a concentrated time frame). These dimensions dynamically influence one another to shape the effective biological stimulation delivered within a therapeutic window. The central construct, “Effective Intensity,” emerges from the multiplicative interaction of these components rather than from therapy volume alone, thereby clarifying distinctions between intensity, duration, frequency, and total dose. This framework provides an operational structure for designing and interpreting future neurorehabilitation trials.

Figure 2 presents a conceptual schematic illustrating the multidimensional structure of rehabilitation intensity and the interactions among its core components.

### 2.4 Distinguishing intensity from duration, frequency, and volume

The revised definition explicitly separates intensity from constructs commonly confounded with it:

- Duration = total time spent in therapy;
- Frequency = number of sessions per week;
- Volume = frequency × duration;
- Intensity = density and potency of stimulation within a given time unit. This distinction is essential because two interventions with identical volumes can elicit dramatically different neuroplastic effects when the intensity differs—a phenomenon consistently demonstrated in CIMT protocols (14), high-intensity interval training (15), and intensive upper limb boot camps (8).

### 2.5 Intensity as a neurobiological rather than administrative construct

Emerging neurophysiological evidence suggests that intensity must be conceptualised primarily as a biological dose, not an administrative metric. Motor cortex remapping, synaptic strengthening, and proprioceptive integration display threshold-dependent activation patterns that correlate with the rate and magnitude of stimulation, rather than with time alone (16–18). This interpretation aligns with theoretical models of use-dependent plasticity, which demonstrate that stimulation delivered below a minimal activation threshold fails to initiate molecular cascades necessary for adaptive change (16). Thus, even prolonged therapy delivered at low density may remain biologically subtherapeutic, whereas briefer but high-intensity protocols may exceed thresholds required for meaningful reorganisation.

### 2.6 Clinical measurability across resource settings

To enable global applicability—including in low-resource environments—each dimension of intensity must be measurable without advanced technology:

- Repetition density can be quantified manually using handheld counters or therapist logs;
- Effort and engagement can be rated using structured behavioural scales adaptable to clinical and community contexts;
- Neurophysiological load may be approximated through task difficulty grading, Borg scales, or proprioceptive challenge levels;
- Temporal compression is calculated from therapy schedules. This approach facilitates transposition across both high-income and low-income health systems, supporting cross-cultural comparisons and enabling family-based intensity amplification models developed in African contexts (9).

### 2.7 Implications for research and measurement standards

A unified operational definition allows:

- Standardisation across future RCTs;
- Re-analysis of existing datasets using intensity metrics;
- More accurate modelling of dose–response relationships;
- Harmonisation with technology-enabled intensity quantification (e.g., wearable sensors), without making the core definition dependent on technological access. This definition therefore frames intensity as a quantifiable, testable, and clinically deployable construct foundational for the theoretical models elaborated in Section 4.

## 3 Contemporary evidence synthesis (2018–2025)

Understanding the relative contribution of rehabilitation intensity versus duration requires an examination of contemporary evidence from clinical trials, mechanistic studies, and cross-cultural implementation models. Over the past decade, research has progressively shifted away from time-based prescriptions toward biologically grounded dose–response frameworks. This evolution has produced a growing number of trials in which intensity—rather than overall duration—constitutes the primary manipulated parameter (19–21).

To provide an empirical foundation for the theoretical models introduced in Section 4, we synthesised findings from key studies published between 2018 and 2025, focusing on interventions that directly contrast intensity-driven strategies with duration-dominant or volume-matched paradigms. A structured comparison of these trials is presented in Table 1, which highlights their methodological features, dosing organisation, and recovery trajectories.

**Table 1:** Comparative characteristics and outcomes of contemporary intensity-focused versus duration-focused stroke rehabilitation trials (2018–2025).

Study / year	Design/sample	Phase	Intervention	Intensity vs duration	Outcomes	Key findings
Queen Square Programme (8)	Prospective cohort; *n* = 224	Chronic	90 h/3 wks (6 h/day) massed task practice	Same total hours as usual care; *3 × higher density*	ARAT, WMFT, FMA-UE	Greater gains; *intensity predicts outcome better than duration*.
Boyne et al. (15) (HIIT)	RCT; *n* = 40	Subacute	HIIT (70–85% HRpeak) vs. moderate cycling	*Identical duration*; intensity altered	VO₂ peak, gait speed	HIIT superior; intensity drives cardio–motor adaptation.
High-Repetition UL Training (Birkenmeier/Lang)	Repeated cohorts; n ≈ 30	Chronic	300–600 reps/session vs. 30–50 usual	*Higher repetition density*; same duration	FMA-UE, MAL	High-repetition training consistently superior.
CIMT Compressed vs Distributed (2018–2024)	Umbrella review	Subacute and chronic	3 h/day × 2 wks vs. 1 h/day × 6 wks	*Equal volume*; compressed intensity more effective	WMFT, FMA-UE	Compressed CIMT consistently superior.
Robotic Intensification Trials (2019–2024)	Multicentre RCTs; n > 350	Subacute	45–60 min/day end-effector massed practice	*High repetition density* despite short sessions	FMA-UE, ADL	Repetition density—not duration—best predictor of gains.
High-Frequency Telerehabilitation (2020–2024)	RCTs; *n* ≈ 700	Subacute	App-guided 60–90 min/day massed tasks	*Higher task frequency*; standard duration	ARAT, grip, adherence	Digital high-density practice outperforms low-frequency home care.
Amanzonwé (26) (Benin)	RCT; *n* = 165	Acute	HIIT + PT vs. sham cycling	Same duration; *intensity modified*	mRS, 6MWT, gait speed	High-intensity arm superior; safe in acute phase.
Franco–Cameroonian Intensive Model (9)	Comparative cohort; *n* = 78	Acute → chronic	2–4 h/day multimodal high-density therapy + family continuation	*Very high task density*, limited duration	FMA-UE, BI, gait	Faster recovery despite limited resources; intensity central.
Cogni-Famille Protocol (27)	Retrospective; *n* = 62	Subacute/chronic	4–6 h/day proprioceptive + cognitive + manual therapy	*Intensity amplified by family training*	FMA-UE, MMSE, FIM	Outcomes comparable to European intensive centres.
Intensive Neuroplasticity Framework (10)	Integrative evidence synthesis combining PubMed-indexed literature, clinical observational data, and cross-context RCT comparisons	Subacute/chronic	3–5 h/day multimodal neurofunctional stimulation integrating motor, sensory, cognitive and family-mediated practice	Higher repetition density and temporal compression vs. duration-based European protocols	FMA-UE, gait speed, Barthel Index, patient activation	Unified neuroplasticity-driven model; faster functional gains when intensity thresholds, critical windows and therapeutic momentum are synchronised; demonstrates cross-cultural scalability of intensity-based rehabilitation

### 3.1 Evidence from high-intensity, high-density rehabilitation models

One of the most influential contributions in this period is the Queen Square Upper Limb Programme, which demonstrated that a threefold increase in task density, delivered within a short therapeutic window (3 weeks), produced significantly greater functional gains than standard care despite equivalent total therapy hours (8). These findings confirmed that temporal compression of repeated goal-oriented tasks, rather than cumulative duration itself, is a key determinant of motor recovery.

Similarly, multiple replications of high-repetition upper limb training have shown that sessions with 300–600 repetitions—versus the 30–50 typically observed in conventional practice—yield superior outcomes even when session duration is identical (22). These studies reinforce repetition density as a central driver of neurofunctional change.

### 3.2 Modulation of intensity independent of duration

High-intensity interval training (HIIT) trials in subacute and chronic stroke provided further evidence that manipulating physiological load, while keeping duration constant, enhances gait speed, cardiorespiratory fitness, and walking endurance. These results parallel findings in motor rehabilitation, where metabolic and physiological load significantly modulate neuroplastic responsiveness.

Robotic upper limb therapy trials published between 2019 and 2024 also contributed to this evidence base. Although session durations were typically short (45–60 min), the very high repetition density achievable through end-effector devices was consistently associated with measurable improvements in FMA-UE and ADL outcomes (23). The predictive power of repetition density over time spent underscores the biological relevance of intensity.

### 3.3 Intensity compression in constraint-induced movement therapy

Constraint-induced movement therapy (CIMT) has long served as a benchmark for intensity-focused rehabilitation. Recent umbrella reviews comparing compressed versus distributed formats demonstrated that short-term, high-intensity protocols were consistently superior to distributed low-density versions, despite identical total hours of therapy (24). These results confirm that time compression and increased repetition density amplify neuroplastic cascades beyond what duration alone can achieve.

### 3.4 Digital intensification and telerehabilitation (2020–2024)

Advances in digital health have enabled remote delivery of high-frequency task cycles. Multiple RCTs indicate that app-guided telerehabilitation systems, which schedule 60–90 min/day of task-specific massed practice, produce better upper limb outcomes than standard low-frequency home programmes (25). Importantly, although session duration was not increased, task frequency and engagement metrics were markedly higher. These findings open pathways for scalable high-intensity rehabilitation, including in low-resource settings.

### 3.5 Evidence from low-resource African contexts

Two key developments strengthen the global relevance of intensity-driven models:

- *The Benin HIIT Trial* (26). A large, randomised trial demonstrated that high-intensity aerobic stimulation, when combined with physiotherapy, significantly improved gait speed and mRS scores compared with a low-intensity sham protocol, despite identical duration. These findings highlight the safety and efficacy of intensity manipulation even in acute stroke phases.
- *Franco–Cameroonian Comparative Models* (9, 10). Recent comparative observations from Cameroon and France indicate that high-density multimodal therapy, delivered for 2–4 h/day with strong family participation, generated faster gains than low-frequency, technology-equipped European routines.
- *The same principle emerged in the Cogni-Famille protocol* (27), where family-mediated intensification (4–6 h/day) produced outcomes comparable to European intensive centres. Together, these findings support the hypothesis that intensity-driven neuroplasticity is culturally and contextually generalisable, and not dependent on advanced technology or high-income health systems.

### 3.6 Synthesis

Across all paradigms—from high-income robotic environments to low-resource family-mediated settings—three convergent trends emerge:

1. Intensity consistently outperforms duration, even when total therapy hours are identical.
2. Repetition density and temporal compression are the strongest predictors of improvement.
3. Intensity effects are robust across cultures, delivery methods, and resource levels, indicating that the underlying mechanisms are biological rather than technological or socioeconomic.

These empirical insights form the evidentiary foundation for the three theoretical models articulated in Section 4.

## 4 Integrated theoretical framework

The evidence summarised in Section 3 demonstrates that intensity-driven recovery is a complex, non-linear process emerging from the interaction of threshold-dependent neuroplastic mechanisms, temporally regulated windows of heightened responsiveness, and cumulative momentum generated through dense, goal-directed practice. In response to reviewer recommendations, we propose an integrated theoretical framework comprising three unified, testable models that provide a coherent mechanistic explanation for the superiority of intensity over duration across diverse clinical and cultural contexts.

### 4.1 The Intensity Threshold and Saturation Model (ITSM)

The ITSM posits that neuroplastic change depends on achieving a minimal activation threshold, below which stimulation is insufficient to trigger adaptive responses (16–18). When repetition density, load, and engagement fall below this threshold, even prolonged therapy yields minimal gains, as seen in trials of increased duration without increased intensity (4, 6–8). Above the threshold, neuroplastic processes are activated, as exemplified by high-intensity protocols like the Queen Square Programme (8) and high-repetition training (22). However, beyond an upper saturation point, additional stimulation produces diminishing returns, consistent with metabolic and attentional constraints (23, 24). The ITSM provides a mechanistic basis for why duration alone is an insufficient proxy for biological stimulation.

### 4.2 The Critical Window Synchronization Model (CWSM)

The CWSM synthesises evidence that neuroplastic responsiveness follows a time-dependent curve, with heightened plasticity in the early post-stroke weeks (28–30). Intensity is most effective when synchronised with these critical windows. During the early enhanced plasticity phase (first 4–6 weeks), high-intensity interventions lead to disproportionately large gains, as seen in both African and European contexts (9). In later phases, higher stimulation density is required to achieve equivalent effects. Interestingly, African family-integrated systems like Cogni-Famille naturally exploit these windows by providing continuous stimulation throughout the day (27), explaining how some low-resource settings achieve outcomes comparable to high-tech centers through temporal synchronisation rather than technology alone.

### 4.3 The Therapeutic Momentum and Cascade Model (TMCM)

The TMCM conceptualizes recovery as a momentum-driven process, where each intensive stimulation bout increases the likelihood of further gains, supported by studies of motor learning, reinforcement, and behavioural activation (31–33). Repeated high-density practice builds physiological and cognitive momentum, increasing task automaticity and consistency. Conversely, low-density therapy produces insufficient momentum, leading to inter-session decay and plateauing. The Cogni-Famille and Franco-Cameroonian models demonstrate how family involvement can amplify momentum by maintaining high stimulation rates throughout the day (27). The TMCM highlights the importance of sustained, frequent intensity, beyond isolated clinical sessions.

### 4.4 Integrated interpretation

When combined, these models provide a cohesive mechanistic foundation:

- ITSM explains why intensity is necessary (biological threshold)
- CWSM explains when intensity is most effective (temporal alignment)
- TMCM explains how intensity produces lasting change (momentum)

**Figure 3:**
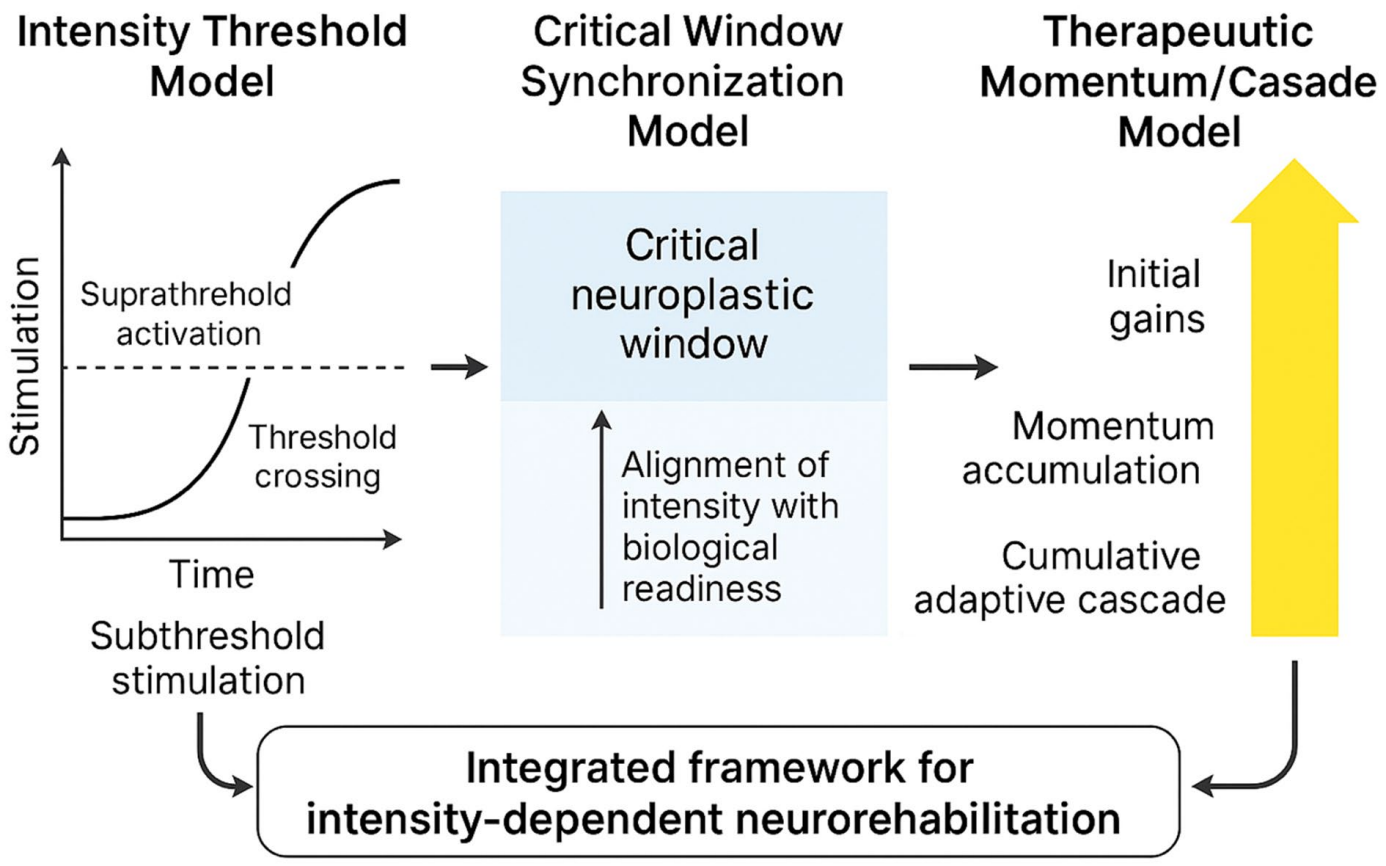
Integrated theoretical framework for intensity-dependent neurorehabilitation: threshold, temporal windows, and therapeutic momentum. The framework integrates three complementary models: (1) The *intensity threshold model*, which posits that neuroplastic reorganisation is initiated only when the rate and magnitude of stimulation exceed a defined biological activation threshold; (2) the *critical window synchronization paradigm*, which conceptualises the interaction between intensity and temporally sensitive phases of heightened neural receptivity, particularly within early post-stroke periods; and (3) the *therapeutic momentum & cascade model*, which describes how high-density, goal-directed stimulation generates cumulative adaptive processes that amplify recovery trajectories over time. Together, these models outline how repetition density, neurophysiological load, engagement–effort dynamics, and temporal compression converge to produce measurable gains in motor and functional outcomes. The framework provides a unified conceptual structure for interpreting empirical findings across diverse clinical and cultural contexts, including high-, middle-, and low-resource environments.

Together, they align neuroscience with clinical practice across diverse global settings, as summarised in Figure 3.

## 5 Mechanistic vs. clinical evidence: a hierarchical interpretation

The preceding sections establish that rehabilitation intensity influences neurofunctional recovery through threshold-dependent mechanisms, temporally sensitive plasticity windows, and momentum-driven behavioural cascades. However, the strength of these claims depends on a rigorous appraisal of the different levels of evidence that contribute to this understanding. Addressing the reviewer’s methodological concerns, this section articulates a clear hierarchical structure distinguishing mechanistic, clinical, and implementation evidence.

### 5.1 Mechanistic evidence (level 1)

Mechanistic evidence elucidates how intensity produces neuroplastic change at the cellular, synaptic, and systems levels. Foundational experiments by Jenkins and Merzenich (18), Nudo et al. (17), Kleim and Jones (16), and subsequent work in rodent and primate models demonstrate three convergent principles:

1. Use-dependent cortical reorganisation requires surpassing an activation threshold. Below this threshold, synaptic potentiation and dendritic sprouting do not occur.
2. Temporal clustering of stimulation enhances neuroplastic yield. Studies show that delivering stimulation in concentrated bouts triggers stronger long-term potentiation than spreading the same number of repetitions over extended durations.
3. Non-linear, cumulative effects govern adaptive responses. Repetition accelerates responsiveness: each bout increases the likelihood of subsequent plastic change, supporting the momentum-based mechanisms modelled in Section 4. These mechanisms provide the biological underpinnings for the Intensity Threshold and Saturation Model (ITSM) and the Therapeutic Momentum and Cascade Model (TMCM), but they do not, in isolation, determine clinical efficacy.

### 5.2 Clinical efficacy evidence (level 2)

Clinical trials examine whether intensity improves functional outcomes in humans. The strongest evidence arises from:

- Large-scale RCTs (e.g., HIIT, robotic therapy, CIMT compressed formats) (23–25);
- Replicated high-repetition upper limb protocols (22);
- The Queen Square Programme (8). Across these trials, three robust findings emerge:

1. Interventions delivering higher repetition density outperform duration-equivalent control conditions.
2. Temporal compression—short, intensive schedules—enhances the rate of functional improvement even when total therapy hours are identical.
3. Metabolic and attentional load modulation independently contributes to functional gains. Clinical efficacy evidence therefore validates the ITSM and TMCM predictions and provides human confirmation of mechanisms first described in animal models.

### 5.3 Clinical effectiveness and real-world: feasibility evidence (level 3)

Effectiveness evidence evaluates whether intensive protocols succeed under real-world conditions, where constraints such as therapist availability, family involvement, and cultural organisation of care matter. This layer includes:

- The Franco–Cameroonian comparative model (9);
- The Cogni-Famille intensive manual therapy system (27);
- African trials such as the Benin HIIT RCT (26). These studies confirm that:

1. Intensity-driven mechanisms are culturally generalisable.
2. High-density stimulation can be achieved without advanced technology when family members participate as intensity multipliers.
3. Outcomes in low-resource systems rival or surpass those achieved in technologically equipped centres. This evidence does not replace mechanistic or efficacy data, but it demonstrates that intensity is implementable and scalable across contexts.

### 5.4 Practical implications of hierarchical distinction recognising these three layers clarifies several conceptual issues

**Figure 4:**
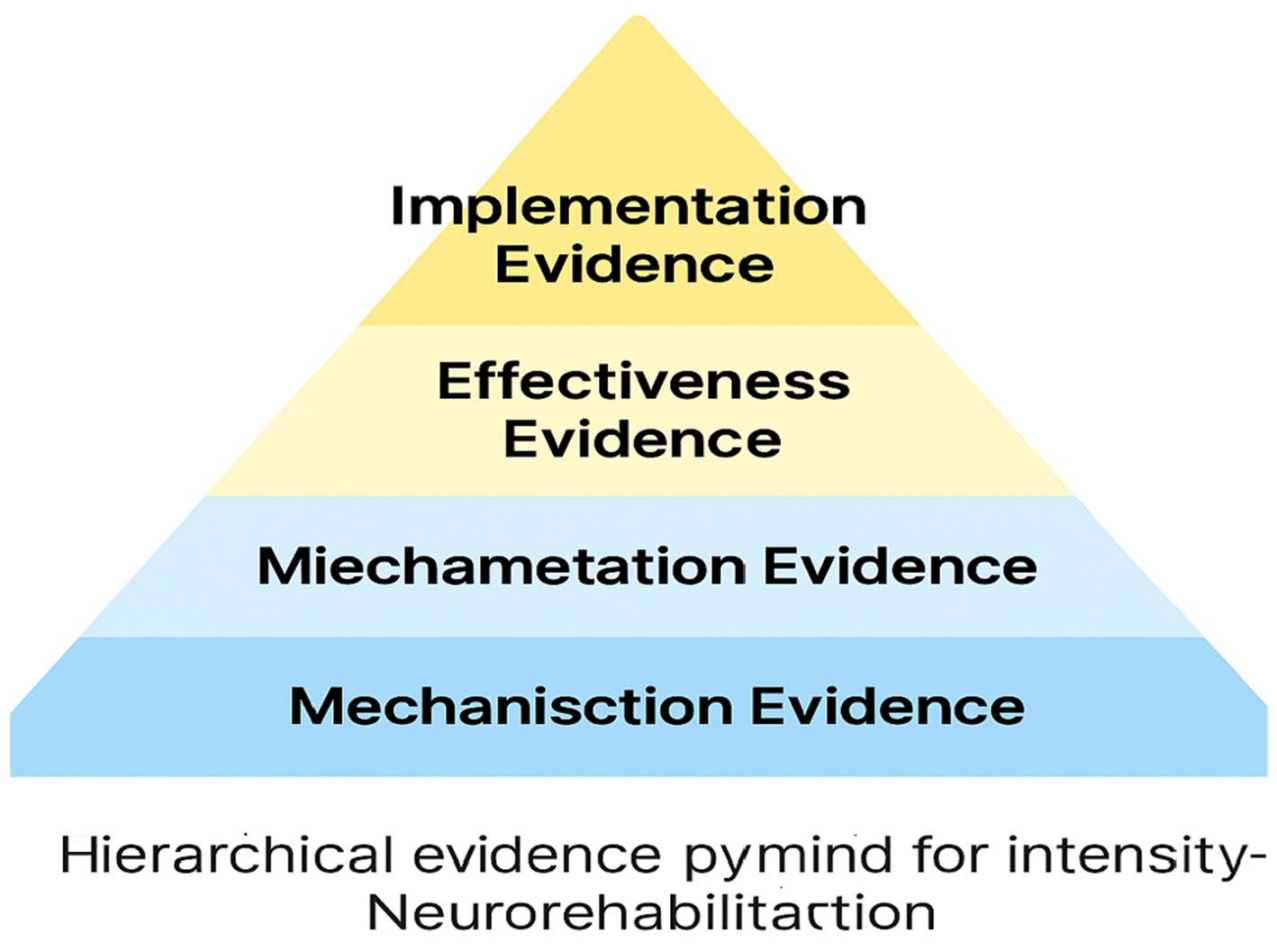
Hierarchical evidence pyramid for intensity-based neurorehabilitation (Mechanistic → Efficacy → Effectiveness → Implementation): bridging theoretical models to empirical outcomes. This integrative framework synthesises three complementary models (Intensity Threshold Model, Critical Window Synchronization Model, and Therapeutic Momentum & Cascade Model) and maps them onto a structured hierarchy of evidence spanning mechanisction, miechametation, effectiveness, and implementation levels. The pyramid illustrates how core components of effective intensity (repetition density, neurophysiological load, engagement-effort dynamics, and temporal compression) interact across different scales of analysis, from animal neurophysiology to human clinical trials and cross-cultural feasibility studies. By aligning theoretical constructs with empirical findings, this framework provides a unified explanatory model for intensity-dependent plasticity and recovery, addressing a key gap identified by reviewers. The integration of mechanistic insights, dose–response data, efficacy metrics, and real-world implementation evidence within a single conceptual architecture enhances the translational utility and predictive power of intensive rehabilitation paradigms. This synthesis guides hypothesis generation, clinical decision-making, and research prioritization, setting the stage for a new era of precision neurorehabilitation centered on optimising intensity parameters for individual patients. The hierarchical evidence pyramid thus serves as a unifying lens to interpret past findings, design future studies, and harness the potential of intensive training to maximise neuroplastic gains and functional outcomes across diverse clinical populations and contexts.

- Mechanistic evidence explains why intensity matters.
- Clinical efficacy evidence demonstrates that intensity improves outcomes.
- Real-world effectiveness evidence shows how intensity can be delivered sustainably across settings (see Figure 4).

## 6 Cross-cultural implementation and family-based intensification

The models and evidence reviewed so far indicate that rehabilitation intensity functions as a biological driver of recovery rather than as a mere organisational parameter. However, its clinical impact ultimately depends on whether high-intensity protocols can be implemented sustainably across health systems with markedly different resources, cultures, and professional configurations. Cross-cultural experiences from Europe and sub-Saharan Africa have particular heuristic value because they expose the underlying mechanisms of intensity in settings where technology, staffing, and financing differ radically (9, 26, 27).

### 6.1 Intensity beyond technology: lessons from low-resource systems

In high-income environments, intensity is often pursued through robotics, virtual reality, or specialised inpatient programmes. By contrast, African models have emerged in contexts with limited equipment, fewer therapists per capita, and high out-of-pocket costs. Comparative Franco–Cameroonian experiences suggest that therapeutic density can be achieved without advanced devices, provided that three conditions are met: (1) extended daily exposure to task-specific practice; (2) systematic task progression within and across sessions; and (3) mobilisation of family members as co-therapists (9). These configurations demonstrate that intensity is fundamentally organisational and relational before it is technological.

### 6.2 Family as intensity multipliers

The Cogni-Famille protocol operationalises this organisational principle by training relatives to deliver large volumes of proprioceptive, cognitive, and manual stimulation throughout the day (27). From the perspective of the Therapeutic Momentum and Cascade Model (TMCM), family members act as momentum amplifiers: they reduce the decay between formal sessions, maintain high repetition density in the home environment, and increase the proportion of waking hours spent above the neuroplastic activation threshold. This approach also modifies the traditional division of labour between professionals and laypersons, entrusting families with a structured, high-intensity regimen that is continuously supervised but not continuously delivered by clinicians.

### 6.3 Cultural organisation of time and practice

Cross-cultural implementation also reveals how cultural norms around caregiving and household organisation interact with intensity. In many African settings, multigenerational households and strong kinship obligations make it feasible for grandmothers, siblings, or neighbours to participate in daily rehabilitation routines. This social architecture naturally supports high temporal compression of practice, provided that the regimen is clearly defined, realistically dosed, and aligned with family routines. By contrast, in some European contexts, patients may have greater access to technology and professional services but fewer hours of informal caregiving.

### 6.4 Safety, burden, and ethical considerations

Implementing intensity across cultures also requires attention to safety and caregiver burden. High-density protocols must respect cardiovascular, orthopaedic, and cognitive safety limits, particularly when applied in the acute phase or in patients with multimorbidity (26). Similarly, family-mediated programmes must avoid unsustainable workloads, emotional exhaustion, or the inadvertent transfer of professional responsibilities without adequate training and follow-up. From an ethical standpoint, intensity should not be interpreted as a justification for overloading families or underfunding professional services. Instead, cross-cultural models suggest that the optimal configuration is a calibrated partnership, in which professionals design, monitor, and periodically adjust high-intensity regimens that families help to implement within their own cultural and economic realities.

### 6.5 Implications for global rehabilitation policy

The cross-cultural portability of intensity-focused paradigms has several implications for policy and service design:

1. Intensity as a core quality indicator: Health systems should monitor not only therapy minutes but also repetition density and temporal compression as key indicators of rehabilitation quality.
2. Structured family training as a standard component of care: Programmes should incorporate structured curricula that transform relatives into safe, effective intensity multipliers.
3. Context-sensitive models of precision rehabilitation: Precision rehabilitation should be conceptualised not only in terms of biomarkers and algorithms, but also in terms of social ecology.
4. Bridging high- and low-resource systems: High-income settings can learn from African models how to better exploit home-based and family-mediated intensity, while African systems can adapt selected technological tools that objectively quantify repetitions, engagement, or gait cycles without replacing relational care.

By reframing intensity as a globally adaptable organisational principle, this section closes the conceptual loop between mechanistic models, clinical trials, and real-world feasibility. It also prepares the ground for the next section, which formalises the research gaps and testable hypotheses that emerge from this cross-cultural, multilevel synthesis.

## 7 Research gaps and priority hypotheses

Despite growing convergence toward intensity-focused paradigms, several fundamental questions remain unresolved. These gaps concern (1) the precise dose–response architecture of rehabilitation intensity, (2) inter-individual variability in responsiveness, (3) temporal personalisation of intensive protocols, (4) objective quantification of intensity in real-world settings, and (5) the translation of intensity indices into service-level quality metrics. Addressing these questions requires explicitly testable hypotheses derived from the models proposed in Sections 2–4.

### 7.1 Unresolved dose–response architecture for intensity

Current evidence suggests that “more intensive” rehabilitation is generally associated with better outcomes, yet the field still lacks a robust, domain-specific mapping of the intensity–response curve. Most trials contrast “usual care” with a higher-dose intervention, without systematically varying intensity across multiple graded levels or separating intensity from total volume (34, 35).

Priority questions include:

1. What is the minimal effective intensity threshold (I_min) for different functional domains and severities?
2. Where is the saturation point (I_sat) beyond which additional intensity yields diminishing or even negative returns?
3. How do volume and intensity interact?

*Priority Hypothesis 1*. For each combination of stroke phase, impairment profile, and functional domain, there exists a quantifiable intensity threshold (I_min) above which gains per unit time are significantly greater than those obtained with time-matched, subthreshold interventions, independent of total volume.

Testing this hypothesis requires multi-arm trials that manipulate I explicitly while keeping frequency and duration constant.

### 7.2 Phenotype-specific responsiveness to intensity

Accumulating evidence indicates that not all patients respond equally to intensified therapy. Retrospective analyses and recent meta-analyses suggest that time post-stroke, baseline severity, corticospinal tract integrity, and comorbidities modulate responsiveness to high-intensity protocols (8, 35–37). However, these determinants have not yet been integrated into a coherent “intensity responsiveness phenotype” framework.

*Priority Hypothesis 2.* Patients can be classified into distinct intensity responsiveness phenotypes based on a combination of neurological biomarkers, time since stroke, and environmental variables. Within each phenotype, intensity–response curves will display characteristic shapes.

This hypothesis calls for prospective stratified trials and secondary analyses of existing datasets, using the operational intensity index proposed in Section 2.

### 7.3 Temporal personalisation of intensity windows

The Critical Window Synchronization Model (CWSM) posits that intensity is most effective when delivered during periods of heightened plastic potential. While early enhanced plasticity within the first weeks post-stroke is well described, contemporary data still provide limited guidance on the optimal timing, ramp-up speed, and tapering of intensity (8, 28–30, 36).

*Priority Hypothesis 3.* For medically stable patients, early front-loaded intensity (above I_min, delivered within the first 4–6 weeks) produces superior long-term functional outcomes and reduced chronic disability compared with delayed intensification protocols matched for total volume.

This hypothesis can be tested through phase-specific RCTs that contrast early versus delayed intensity, while carefully monitoring safety endpoints and spontaneous recovery trajectories.

### 7.4 Objective quantification of intensity in real-world and family-mediated contexts

Although Section 2 proposed a clinically measurable intensity index, most real-world services still rely on crude proxies. Recent advances in wearable sensors, digital biomarkers, and telerehabilitation platforms offer opportunities to continuously quantify repetitions, movement quality, and engagement both in clinics and at home (38–42). Yet these technologies have been only partially integrated into intensity research, particularly in low-resource settings.

*Priority Hypothesis 4.* In both high- and low-resource environments, integrating low-cost wearable sensors and simple digital interfaces into family-mediated protocols will (a) allow reliable estimation of rehabilitation intensity and (b) yield functional outcomes non-inferior to centre-based intensive programmes, at a lower cost per unit of functional gain.

Addressing this hypothesis requires hybrid implementation–effectiveness studies comparing sensor-augmented family programmes with conventional institutionally delivered intensity.

### 7.5 Health-system and policy-level gaps: intensity as a quality indicator

At the health-system level, most policy documents and reimbursement schemes continue to index rehabilitation quality on duration rather than on biologically relevant intensity metrics. Even recent health technology assessments and telerehabilitation evaluations rarely report standardised intensity indices, despite acknowledging the central role of dose (34, 35, 43).

*Priority Hypothesis 5*. Across stroke rehabilitation services, higher unit-level intensity KPIs will be independently associated with better patient-level functional outcomes and reduced long-term disability, after adjustment for case-mix, staffing ratios, and technological infrastructure.

Testing this hypothesis requires large-scale health-services research linking routinely collected clinical data, intensity metrics, and long-term outcomes, ideally across multiple countries and resource strata.

## 8 Clinical and policy implications for precision rehabilitation

The framework developed in this Perspective has two major implications. First, rehabilitation intensity must be conceptualised as a biological dose rather than an administrative quantity, consistent with the principles of use-dependent plasticity (16–18). Second, precision rehabilitation should integrate intensity metrics alongside neurobiological, functional, and contextual variables, in line with emerging standards in stroke recovery science (13, 19).

### 8.1 From session minutes to biologically grounded dosing

In routine practice, prescription and documentation remain dominated by duration. Yet, evidence summarised in Sections 2–4 shows that duration is an unreliable surrogate for the neurobiological dose required to exceed activation thresholds (16–18). This has three practical consequences:

1. systematic estimation of repetition density and active time rather than gross duration, consistent with dose–response analyses (20, 33);
2. explicit titration of load and engagement to remain above I_min, supported by metabolic-load studies from HIIT and intensive motor practice (23);
3. progressive adoption of simple measurement tools, anticipating recommendations for standardised intensity reporting frameworks (34).

These adjustments reframe clinical documentation away from “45 min of therapy” toward “biologically meaningful intensity delivered within safe physiological limits.”

### 8.2 Embedding intensity in individualised care pathways

The ITSM, CWSM, and TMCM converge toward an individualised dose–response logic consistent with contemporary recommendations for personalised rehabilitation (8, 13). In practice, this implies:

- preliminary estimation of the patient’s intensity responsiveness phenotype, grounded in biomarkers and contextual variables (8, 35–37);
- setting patient-specific intensity targets (I_min and I_sat) for each domain, applying evidence from graded-intensity trials (34, 35);
- adapting the temporal delivery profile according to stroke phase and plasticity windows (28–30);
- coordinating institutional and family-mediated practice to maintain therapeutic momentum (27, 31–33).

This approach aligns biological potential with social ecology, rather than relying exclusively on technological sophistication.

### 8.3 Training and professional development

Despite robust evidence linking intensity to outcomes (8, 22–25), formal training in dose–response principles remains limited in most curricula. Educational reforms should integrate:

- foundational neuroplasticity mechanisms (16–18, 28–30);
- applied dosing examples from high-intensity trials (8, 22–25);
- structured competencies for supervising family-mediated intensive programmes (27);
- ethical considerations related to safety, fatigue, and caregiver burden (26).

These competencies are essential for transforming intensity from an exceptional feature of a few centres into a routine standard of care.

### 8.4 Service organisation and funding models

At the health-system level, most funding schemes index reimbursement on duration, not on biologically relevant intensity metrics, despite the clear superiority of intensity demonstrated in multiple trials (8, 22–25, 34, 35). Integrating intensity indicators into service dashboards and reimbursement formulas would align incentives with biological mechanisms instead of administrative constraints. In low-resource systems, recognising intensity as a quality indicator supports investment in staff, training, and basic measurement tools, and prevents the underestimation of the true societal contribution of family-mediated practice (9, 26, 27).

### 8.5 Integration with digital health and data infrastructures

Digital health offers an opportunity to embed intensity metrics into routine care. Recent telerehabilitation and wearable sensor studies demonstrate how repetitions, active minutes, and engagement can be objectively captured and fed back to clinicians (25, 38–42). Integrating such metrics into electronic health records and stroke registries would facilitate:

- large-scale testing of intensity KPIs (Priority Hypothesis 5);
- cross-centre benchmarking;
- dose–response modelling across different resource environments.

Whether through sensor-rich platforms in high-income settings or simplified mobile applications in low-resource contexts, the goal is the same: to align system-level measurement with biological mechanisms of recovery (34, 38–43).

## 9 Limitations

Of the Current Perspective Although this Perspective synthesises mechanistic, clinical, and cross-cultural evidence, several limitations warrant consideration. First, the proposed operational intensity index—while grounded in biological plausibility—requires formal psychometric and clinimetric validation across stroke phases and impairment profiles. Recent measurement studies confirm the feasibility of multi-dimensional dosing indices but highlight variability in construct stability across contexts (44, 45). Second, the synthesis relies on heterogeneous trials in which intensity is manipulated through different mechanisms. Standardisation remains incomplete and may limit direct comparability between studies. Third, real-world observations from low-resource environments, although conceptually valuable, derive primarily from observational cohorts and require replication through controlled pragmatic trials. Finally, the Perspective does not address cost-effectiveness analyses, despite emerging evidence showing that intensity-driven paradigms may reduce long-term disability burden and institutionalisation costs (46).

## 10 Future directions

Based on the integrated framework and research gaps identified in Section 7, several priority avenues emerge.

### 10.1 Validation of intensity metrics

Future trials should incorporate standardised intensity reporting templates and validate dose–response curves using continuous data from sensors, behavioural logs, and engagement metrics, as recently recommended by international rehabilitation consortia (44–47).

### 10.2 Stratified and biomarker-guided intensity trials

Advances in neuroimaging and connectomics offer opportunities for phenotype-based dosing protocols. Biomarkers such as structural connectivity, CST integrity, and functional network modularity have shown promise in predicting responsiveness to high-dose training (48, 49).

### 10.3 Cross-cultural hybrid models

Hybrid intensity models combining structured clinical sessions, wearable-enhanced home practice, and family-mediated routines should be evaluated through multicentre trials across Africa, Europe, and Asia. Implementation science frameworks may help identify contextual moderators (50).

### 10.4 Health-system integration

Intensity-focused KPIs should progressively be incorporated into stroke registries, quality dashboards, and reimbursement frameworks (46, 51, 52). Early policy experiments and methodological consensus papers indicate that standardised, intensity-sensitive measurement systems can enhance clinical outcomes without increasing total healthcare expenditure (51–55). These data reinforce the need for health services to replace session-duration indicators with biologically meaningful metrics such as repetition density, active minutes above threshold, and temporal compression indices.

## 11 Conclusion

Rehabilitation intensity emerges from this synthesis as a biologically grounded, contextually adaptable, and mechanistically coherent determinant of post-stroke recovery. Across multiple layers of evidence—from foundational neuroplasticity experiments to high-intensity clinical trials and family-mediated programmes in low-resource settings—the same principle consistently reappears: recovery is driven not by the number of minutes spent in therapy, but by the density, potency, and organisation of neurofunctional stimulation within those minutes. Intensity, understood as the interaction between repetition density, neurophysiological load, engagement–effort dynamics, and temporal compression, provides a more accurate representation of the therapeutic dose that shapes neural reorganisation.

This Perspective situates rehabilitation intensity within an integrated mechanistic-to-clinical framework that synthesises threshold-dependent activation models, temporally sensitive plasticity windows, and momentum-driven cascades. By articulating this structure and aligning it with empirical evidence, the article reframes intensity as a measurable biological construct rather than an administrative parameter. Such reframing has important implications: it challenges conventional duration-based prescriptions, clarifies the mechanisms through which early intensive therapy yields disproportionate gains, and explains why culturally diverse programmes—such as Franco–Cameroonian models or family-based Cogni-Famille systems—can rival high-technology approaches when repetition density and momentum are sustained.

The next phase of rehabilitation science will require rigorous operationalisation of these concepts. Validating intensity indices, defining phenotype-specific responsiveness profiles, and testing graded intensity interventions across acute, subacute, and chronic phases represent urgent priorities. Likewise, integrating wearable sensors, digital biomarkers, and simplified behavioural metrics will be essential for quantifying intensity objectively across health systems with widely variable resources. From a policy perspective, adoption of intensity-based quality indicators has the potential to recalibrate service organisation, funding mechanisms, and workforce planning around biologically meaningful therapeutic targets.

Finally, advancing rehabilitation intensity as a central pillar of precision rehabilitation requires more than methodological refinement; it requires a cultural shift (56, 57).

## What is known and what is new in this study

### What is known

1. Stroke rehabilitation effectiveness is traditionally assessed based on therapy duration and frequency, despite evidence suggesting intensity (repetition density and potency per unit time) may be a more critical driver of recovery.
2. Animal models and foundational neuroplasticity research demonstrate that use-dependent brain remodeling requires stimulation above minimum thresholds, with higher intensity yielding greater plastic changes.
3. Constrained-induced movement therapy (CIMT) and some high-dose clinical trials suggest massed practice paradigms can outperform distributed lower-intensity sessions despite equal total therapy hours.
4. Neurorehabilitation trials and clinical practice continue to emphasise total treatment time over repetition density and intensity parameters like active minutes, task-specific practice, and progressively challenging exercise.

### What is new

1. This study proposes a rigorous operational definition of rehabilitation intensity, integrating repetition density, neurophysiological load, engagement, and temporal compression, enabling standardised measurement across trials and settings.
2. It synthesises convergent evidence from animal neuroplasticity experiments, imaging studies, motor learning research, high-intensity randomised controlled trials (RCTs), and cross-cultural observational data into three novel mechanistic models: the Intensity Threshold & Saturation Model, Critical Window Synchronization Model, and Therapeutic Momentum & Cascade Model. These models explain how intensity-dependent plasticity operates at physiological and behavioural levels.
3. The analysis reveals that African low-resource, family-integrated rehabilitation paradigms achieve comparable or superior outcomes to technology-driven high-resource models by harnessing temporal synchronisation with critical early plasticity windows and maintaining high-frequency stimulation throughout the day. This challenges assumptions that advanced technology is essential for intensive rehabilitation.
4. The study articulates an intensity-centered framework for precision rehabilitation, proposing to stratify patients into “intensity responsiveness phenotypes” based on neurobiological, clinical and contextual factors and personalize dose–response targets accordingly. This moves beyond one-size-fits-all duration prescriptions.
5. It identifies priority research gaps, including: validation of intensity indices; biomarker-guided responsive subgroup definition; head-to-head comparison of front-loaded intensive versus gradually distributed therapy; hybrid models integrating clinic, home and community-based intensification; and health system policy shifts to incentivize density over duration.
6. The synthesis reframes neurorehabilitation as a threshold-dependent, time-sensitive, momentum-driven process rather than a linear, duration-based service. This aligns clinical practice with the biological reality of neuroplastic adaptation, applicable across cultures and health system resources. Collectively, these insights set the stage for a new era of precision rehabilitation centered on optimising intensity for each patient’s unique profile.

## Data Availability

The original contributions presented in the study are included in the article/supplementary material, further inquiries can be directed to the corresponding author.
